# Cytocompatibility and antibacterial activity of nanostructured H_2_Ti_5_O_11_·H_2_O outlayered Zn-doped TiO_2_ coatings on Ti for percutaneous implants

**DOI:** 10.1038/s41598-017-13954-4

**Published:** 2017-10-24

**Authors:** Lan Zhang, Juan Zhang, Fang Dai, Yong Han

**Affiliations:** State-key Laboratory for Mechanical Behavior of Materials, Xi’an Jiaotong University, Xi’an, 710049 China

## Abstract

To improve skin-integration and antibacterial activity of percutaneous implants, the coatings comprising an outer layer of H_2_Ti_5_O_11_·H_2_O (HTO) nanoarrays and an inner layer of microporous Zn-doped TiO_2_ were fabricated on Ti by micro-arc oxidation (MAO) followed with hydrothermal treatment (HT). During HT process, a large proportion of Zn^2+^ migrated out from TiO_2_ layer. TiO_2_ reacted with OH^−^ and H_2_O, resulting in the nucleation of HTO. The nuclei grew to nanoplates, nanorods and nanofibres with HT process prolonged. Simultaneously, the orientation of nanoarrays changed from quasi-vertical to parallel to substrate. Compared to Ti, adhesion and proliferation of fibroblasts were enhanced on as-MAOed TiO_2_ and HTed coatings. The phenotype, differentiation and extracellular collagen secretion were obviously accelerated on vertical nanorods with proper interspace (e.g. 63 nm). HTed coatings showed enhanced antibacterial activity, which should be ascribed to the nano-topography of HTO.

## Introduction

Titanium and its alloys are widely applied for osseointegrated percutaneous implants due to good mechanical properties and biocompatibility. Unfortunately, Ti lacks antibacterial activity and is bio-inert. The biological sealing of underlying dermis and Ti surface is always weak. Some failure modalities, such as marsupialization, avulsion and infection, have been reported^1^. When Ti implants penetrate skin as foreign bodies, fast skin-implant integration is essential^2^. Fibroblasts play a key role in formation of a dermal layer during the integration process. In early stage of skin healing, fibroblasts are activated, proliferate and switch to a more fibrotic phenotype (characterized with connective tissue growth factor (CTGF)^3^. Simultaneously, they secret collage to synthesize a matrix to provide structural support for wound. Then, fibroblasts express alpha smooth muscle action (α-SMA) and differentiate to a more contractile phenotype, indicating the beginning of officially remolding phase^4–6^. So far, hundreds of coating enhancing bone integration of Ti have been reported. However, relatively fewer coatings are prepared to improve the response of skin-related cells, and they are fibronectin^2^, TiO_2_^7^, hydroxyapatite^8^ and laminin^9^ *et al*. Natural dermis mainly consists of collagen nanofibrils and proteoglycan, and this nanofibrous framework is ideal for the responses of skin-related cells^10,11^. Alkali-heated treatment can directly fabricate nanostructured coatings (e.g. nanofloc, nanorod or nanofiber) on Ti. Unfortunately, the modified surface layers usually have poor adhesions to substrates^12–14^. Recently, a hybrid process of MAO and HT has been used to fabricate nanorod/nanofiber hydroxyapatite or H_2_Ti_5_O_11_·H_2_O on microporous TiO_2_. These hierarchical coatings efficiently affect the osteogenic behaviors of cells (e.g. osteoblast, mesenchymal stem cell) *in vitro* and osteointegration *in vivo* ^15–18^. Nevertheless, whether H_2_Ti_5_O_11_·H_2_O nanoarrays can adhere tightly to Ti substrates and be used on Ti percutanous implants to enhance the behavior of fibroblasts have not been explored.

About the reduction of implant-associated infections, an effective way is to endow the surface of implant with antibacterial activity. Zn has beneficial effect during wound healing^19–22^ and has a broad-spectrum antibacterial activity^23^. Moreover, some phenomena suggest that nano-topography of surface can affect bacterial response. For example, Puckett *et al*.^24^ explored that the nanofeature properties (organization and shape) affect the number of bacteria attached to surfaces. Yee *et al*.^25^ reported that stress caused by nanostructures with precisely defined geometries (e.g. small, closely spaced nanopillars) can kill *E. coli*. However, some researchers suggest that bacteria have characteristic shapes, and compared with eukaryotic cells, they are much less deformable. When bacteria attach to a surface, they can maintain their shapes, and it will hinder interaction between a bacterium and topographical surface features. It means that bacteria may not react to the topographical features in submicrometre and nanometre^26^. Until now, whether nano-topography can affect bacteria adhesion is still in controversial. In this paper, bilayered coatings comprising an outer layer of H_2_Ti_5_O_11_·H_2_O nanoarray and an inner layer of microporous Zn-doped TiO_2_ were designed on Ti. The microstructures and adhesion strengths of these coatings were studied. The formation process of HTed coatings was discussed. The responses of fibroblasts as well as the activity against gram-positive *S. aureus* and gram-negative *E. coli* were investigated to explore their antibacterial property and skin regeneration potential.

## Results

### Structure of as-MAOed TiO_2_ and HTed coatings

**Figure 1:**
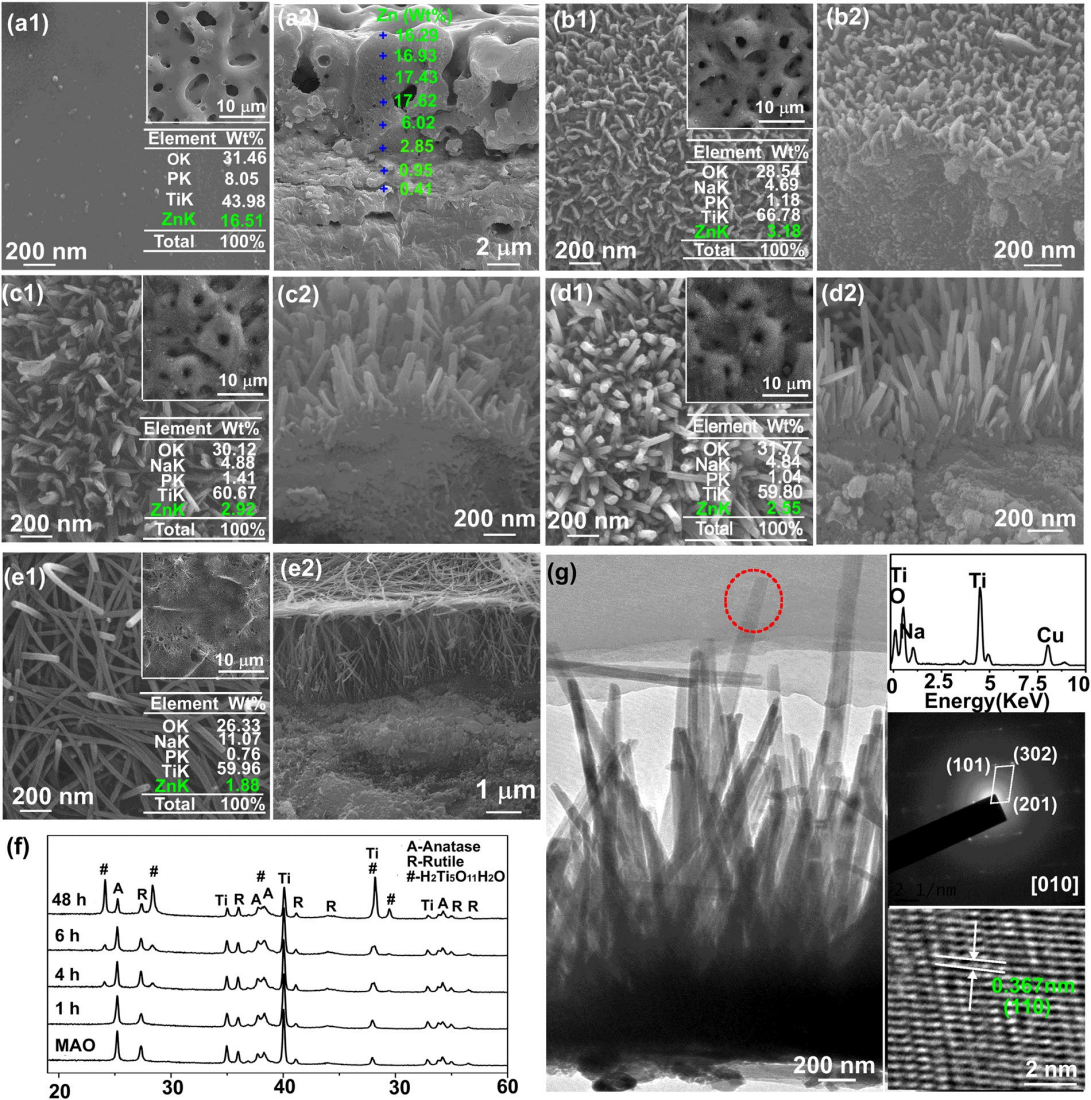
(a1–e1) Surface and (a2–e2) cross-sectional images of coatings: (a1,a2) as-MAOed, (b1,b2) HT1h, (c1,c2) HT4h, (d1,d2) HT6h, and (e1,e2) HT48h, respectively; insets in (a1–e1) show the low magnification images and element contents; (**f**) XRD patterns of MAOed coating before and after HT for different times; (**g**) bright-field image of nanorods scratched from HT6h, insets in (**g**) from top to bottom orderly show the EDS pattern, SAED pattern and HRTEM images.

As-MAOed TiO_2_ is typically nanograined and microporous with pore diameters of 1–4 μm distributing homogeneously over the surface, as shown in Fig. 1(a1). The coating contains O, P, Zn and Ti (Table inserted in Fig. 1(a1)), and average content of Zn is 16.51 Wt%. Thickness of the coating is about 10 μm (Fig. 1(a2)), and the Zn distribution in the cross section at each marked point is represented in Fig. 1(a2), showing that it initially increases with the increased distance from Ti substrate and then reaches a plateau close to the coating surface. After HT for 1 h, a layer of nanoplate nucleate densely on the outermost part of TiO_2_ surface. They are about 80 nm in width, 10 nm in thickness, 45 ± 12 nm in inter spacing (Fig. 1(b1)) and 100 nm in height (Fig. 1(b2)). The HT1h surface contains O, Na, P, Ti and Zn, and the amount of Zn is 3.18 Wt% (Table in Fig. 1(b1)). After HT for 4 h, nanoplates grow into upright-oriented nanorods with a mean width of 80 nm, thickness of 20 nm and length of 200 nm (Fig. 1(c1) and (c2)). The average inter spacing of nanorods is 63 ± 18 nm. The amount of Zn on surface is 2.92 Wt% (Table in Fig. 1(c1)). When HT process further prolonged to 6 h, the width and thickness of nanorods are still about 80 and 20 nm. The mean interrod spacing and length of nanorods increase to 97 ± 18 and 600 ± 100 nm, respectively (Fig. 1(d1) and (d2)). The amount of Zn decreases to 2.55 Wt% (Table in Fig. 1(d1)). The micropores induced by MAO can be observed on HT1h, HT4h and HT6h (Upper insets in Fig. 1(a1–d1)). By further prolonging HT to 48 h, nanorods grow significantly in length and become nanofibers with a mean diameter of 60 nm. The nanofibres are parallel to TiO_2_ layer with an average inter spacing of 105 ± 28 nm (Fig. 1(e1)), and they cover the MAOed micropores (Inset in Fig. 1(e1)) with a thickness of 3 μm (Fig. 1(e2)). The average amount of Zn on HT48h further deceases to 1.88 Wt% (Table in Fig. 1(e1)).

The phase components of samples before and after HT for different hours are shown in Fig. 1(f). As-MAOed TiO_2_ is consisted of anatase and rutile. After HT for 1 h, no new peak is observed, and it should be due to the extreme shortness of nanoplates which cannot be detected by XRD. With increasing HT to 4 h, new peaks at 24.25, 31.25 and 48.40 ° ascribed to H_2_Ti_5_O_11_·H_2_O (JCPDS card no. 44–131) appear, and their intensities enhance with the increased HT time, indicating that nanorods or nanofibres formed on TiO_2_ after HT are H_2_Ti_5_O_11_·H_2_O (Abbreviated as HTO).

For further confirming the microstructure of HTed layer, HT6h surface were scratched and observed by TEM in Fig. 1(g). The bright image shows nanorods with widths of 70–80 nm and lengths of about 600 nm, which are consistent with those in Fig. 1(d). EDX result (upper inset in Fig. 1(g)) of the ring in Fig. 1(g) reveals the nanorod is consisted of Ti, O and Na. Its corresponding SAED pattern (middle inset in Fig. 1(g)) shows distinct spots, indicating high crystallinity of HTO, which is further confirmed by HRTEM in bottom inset in Fig. 1(g). The lattice fringes have an inter-planar spacing of 0.367 nm, which corresponds to the (110) plane of HTO. None of Na or Zn contained compound is detected.

**Figure 2:**
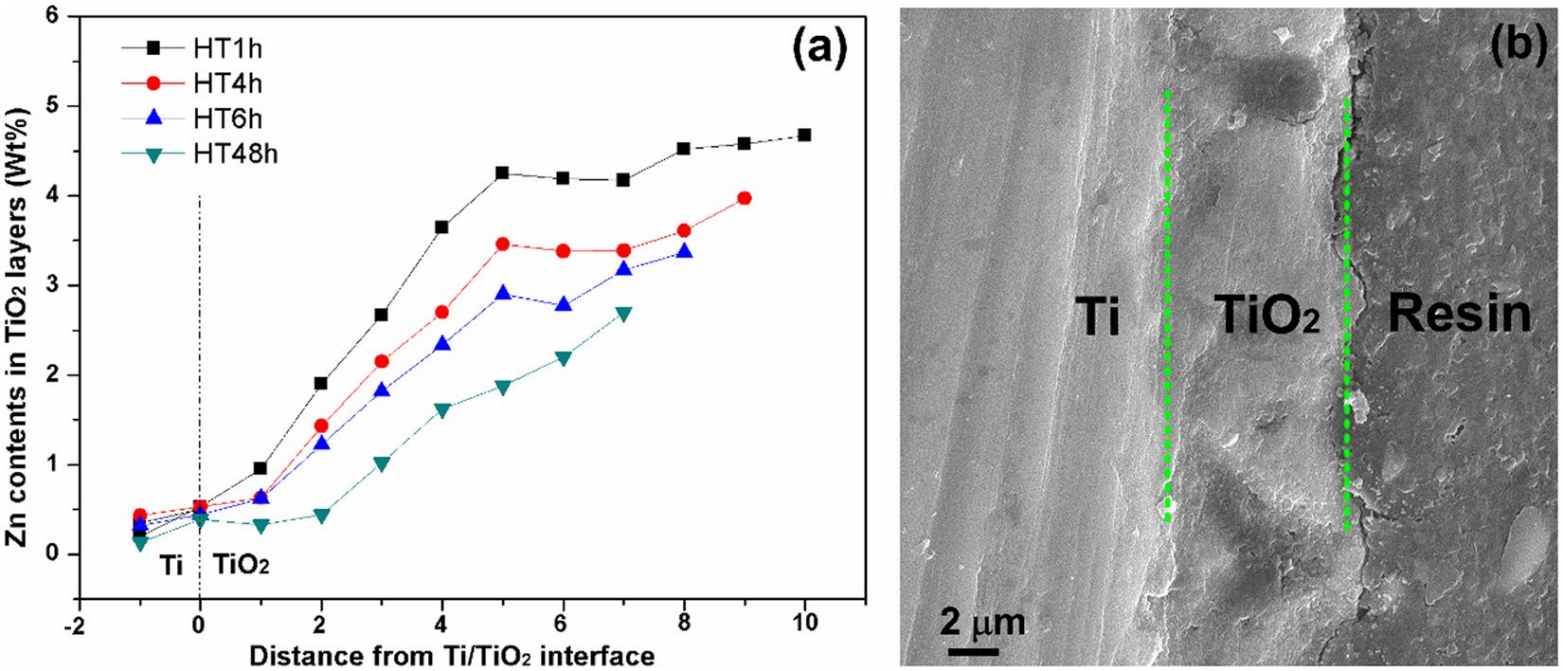
(**a**) Zn profiles in the cross-sections of TiO_2_ layers after HT for different times, (**b**) cross-sectional image of TiO_2_ layer in HT48h.

In order to analyze the formation process of HTed coatings, the cross sections of TiO_2_ layers after HT for different times were examined, and the distributions of Zn in each cross section of TiO_2_ layers are quantified in Fig. 2(a). The contents of Zn in TiO_2_ layers after HT are much less than those in as-MAOed TiO_2_ even though HT was prolonged just for 1 h. With the increased HT time, the thickness of TiO_2_ layer decreases, and at the same distance from TiO_2_/Ti interface, the content of Zn decreases, indicating that TiO_2_ layer is gradually etched and Zn migrates out from the TiO_2_ layer. The thickness TiO_2_ layer in HT48h (Region between the green lines in Fig. 2(b)) is decreased to about 7 μm.

### Bonding strengths of HTxh coatings and wettability

The laser confocal images of coatings indicate the surfaces have similar micro-roughness, and the measured Ra (average roughness) values are 1.00 ± 0.15, 1.00 ± 0.11, 0.95 ± 0.12, 0.90 ± 0.11 and 0.84 ± 0.05 μm for as-MAOed TiO_2_, HT1h, HT4h, HT6h and HT48h, respectively (Supplementary Figure S1).

The critical load of as-MAOed TiO_2_ is 29 ± 3.0 N (Supplementary Figure S2(a)). After HT, the critical loads of coatings do not obviously change (Supplementary Figures S2(b–e)). The amplified views of initial failure caused by Lc (Marked with blue squares) show that delaminations in initial failure areas occurred in the interiors of TiO_2_ layers. They are further confirmed by EDX spectras (insets in Supplementary Figure S2(a–e)), in which O and P are always detected. The wettability of a coating is evaluated by water contact angle. As shown in Supplementary Fig. S2(f), the contact angle is about 55.1°on Ti. After MAO, hydrophilicity of surface is greatly improved, and the contact angle is about 27.5°. The contact angles further decrease after HT, and no obvious difference of contact angle among HTed surfaces is observed. They are almost about 5°, indicating the super hydrophility.

### Zn and Ti release

**Figure 3:**
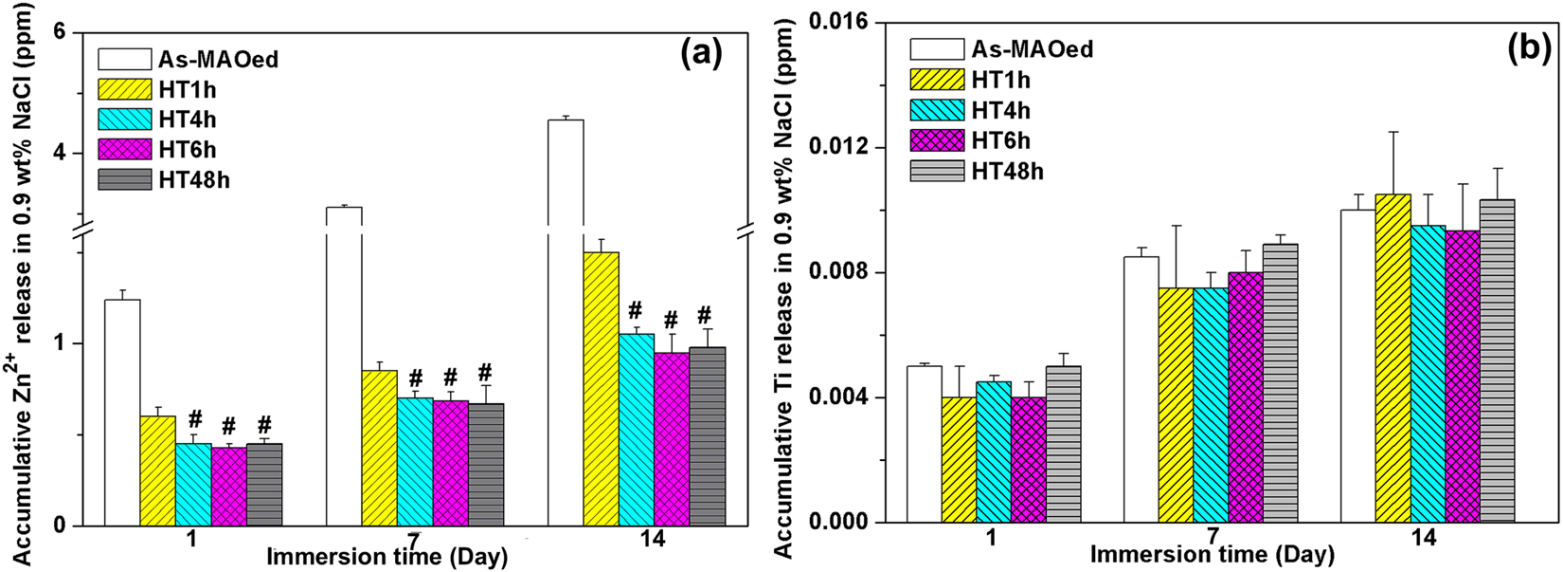
(**a**) Zn and (**b**) Ti concentrations released from different coatings after immersed for 1, 7 and 14 days. ^#^p < 0.01 compared with the HT1h.

The amounts of Zn and Ti released from as-MAOed and HTed coatings were measured, as shown in Fig. 3(a) and (b). With immersion prolonged, cumulated Zn and Ti from each coating all increase. At each immersion time, the amount of Zn released from as-MAOed TiO_2_ is much higher than those from HTed coatings; HT1h releases more Zn than others HTed coatings, whereas highly statistic differences of Zn amount releasing from HT4h, HT6h and HT48h are not observed (Fig. 3(a)). Only traces of Ti are measured at each immersion time, and the released amounts are almost the same for all the samples (Fig. 3(b)). The surface morphologies of HTed coatings after immersion for 14 days were all examined by FESEM and EDX. No obvious changes of morphologies are observed, however, the contents of Zn on surfaces after immersion are slightly lower compared with those before immersion. It indicates that HTO is chemically stable and Zn migrates from coatings into mediums during immersion process.

### Total protein adsorption and *in vitro* fibroblast responses

**Figure 4:**
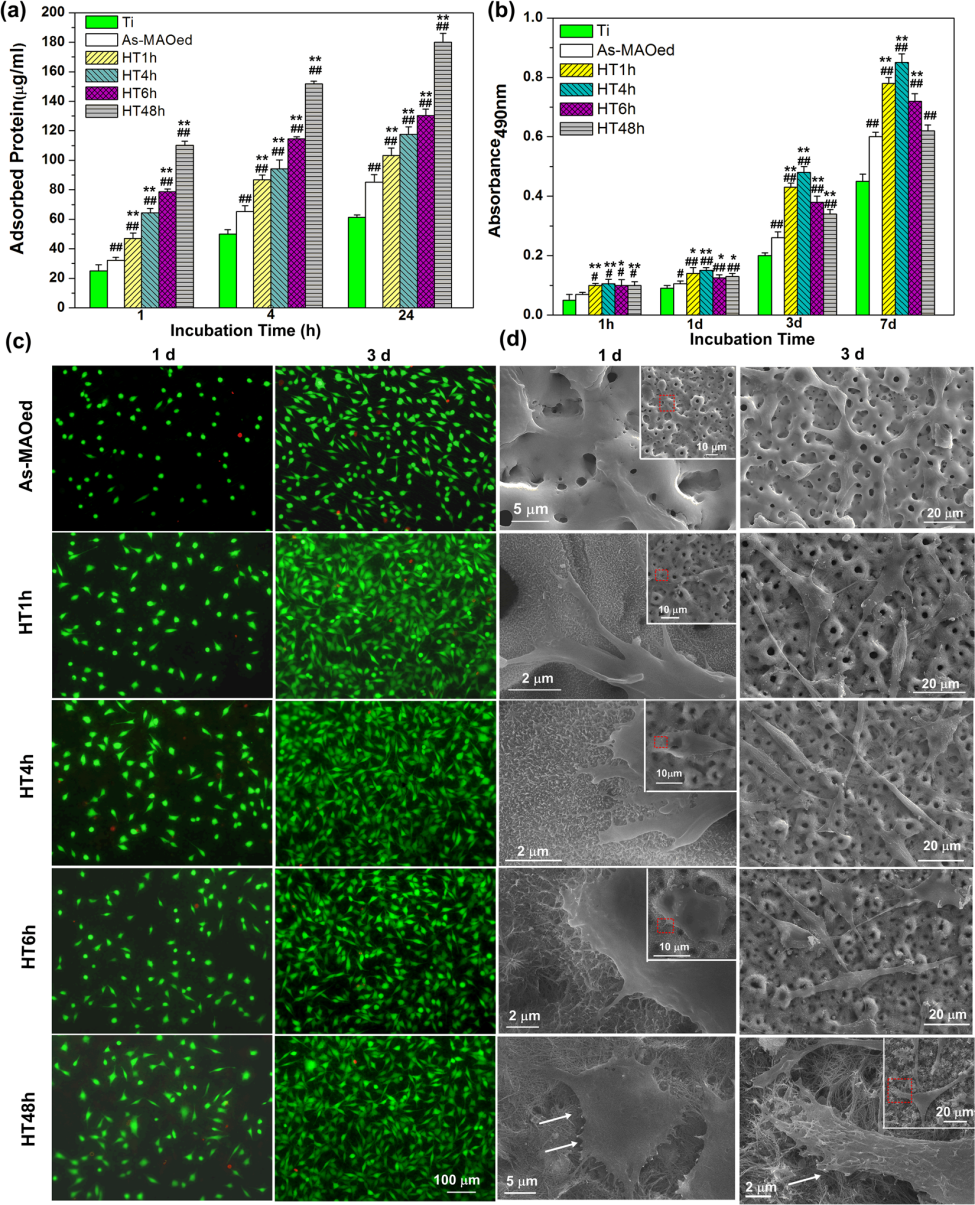
(**a**) Total protein absorbed on different surfaces after incubation for 1, 4 and 24 h; (**b**) MTT assays of cells on different surfaces after incubation for 1 h and 1–7 days; (**c**) Fluorescence images and (**d**) SEM morphologies of cells on different coatings after culture for 1 and 3 days; the insets in (**d**) show corresponding low magnification images. ^#^p < 0.05 and ^##^p < 0.01 compared with Ti, ^*^p < 0.05 and ^**^p < 0.01 compared with as-MAOed TiO_2_.

The amounts of total protein adsorbed on as-MAOed TiO_2_, HTed surfaces and Ti from culture medium after incubation for 1, 4 and 24 h are displayed in Fig. 4(a). With the increased incubation time, the protein amounts from each surface increase, and at each incubation time, they are in the order: HT48h > HT6h > HT4H > HT1h > As-MAOed TiO_2_ > Ti. Figure 4(b) shows the mitochondrial activity of L-929 cultured on different surfaces for 1 h, 1, 3 and 7 d. During the adhesion period (1 h and 1 d), the mitochondrial activity of cells follows the order: HT4h > HT1h > HT48h ≈ HT6h > As-MAOed TiO_2_ > Ti. After 3 or 7 d of incubation, a significant increase of mitochondrial activity of cells on each surface was observed, indicating cell proliferation, and it is in the order: HT4h > HT1h > HT6h > HT48h > As-MAOed TiO_2_ > Ti. The viabilities of cells seeded on as-MAOed and HTed coatings after incubation for 1 and 3 d were also measured by live/dead staining and shown in fluorescence images in Fig. 4(c). Most of the cells (>95%) are stained in green, indicating that they are live, and few of cells are dead and stained in red. At each time point, the live cell numbers on HTed surfaces, especially on HT1h and HT4h, are more than that on as-MAOed TiO_2_. Figure 4(d) shows the morphologies of cells on as-MAOed TiO_2_ as well as HTed surface after incubation for 1 and 3 days. After incubation for 1 d, polygonal cells spread well on the coating surfaces (corresponding insets), and the magnified images of the red squares in the corresponding insets in Fig. 4(d) show more filopodia of cells are observed on the nanoplates and nanorods (e.g. HT1h, HT4h and HT6h). After incubation for 3 d, the number of cells on each surface increases significantly, which is consistent with the staining results in Fig. 4(c). The cells are typically  spindle, and communicate with each other with their elongated finger-like pseudopodium. It is also noticed that on HT48h, most of cells spread well, while a few of cell brims are crimped after 1 and 3 d of incubation (marked with arrows in Fig. 4(d)), indicating some degree of repellence for cell’s spread.

### Intracellular proteins and extracellular collagen secretion

**Figure 5:**
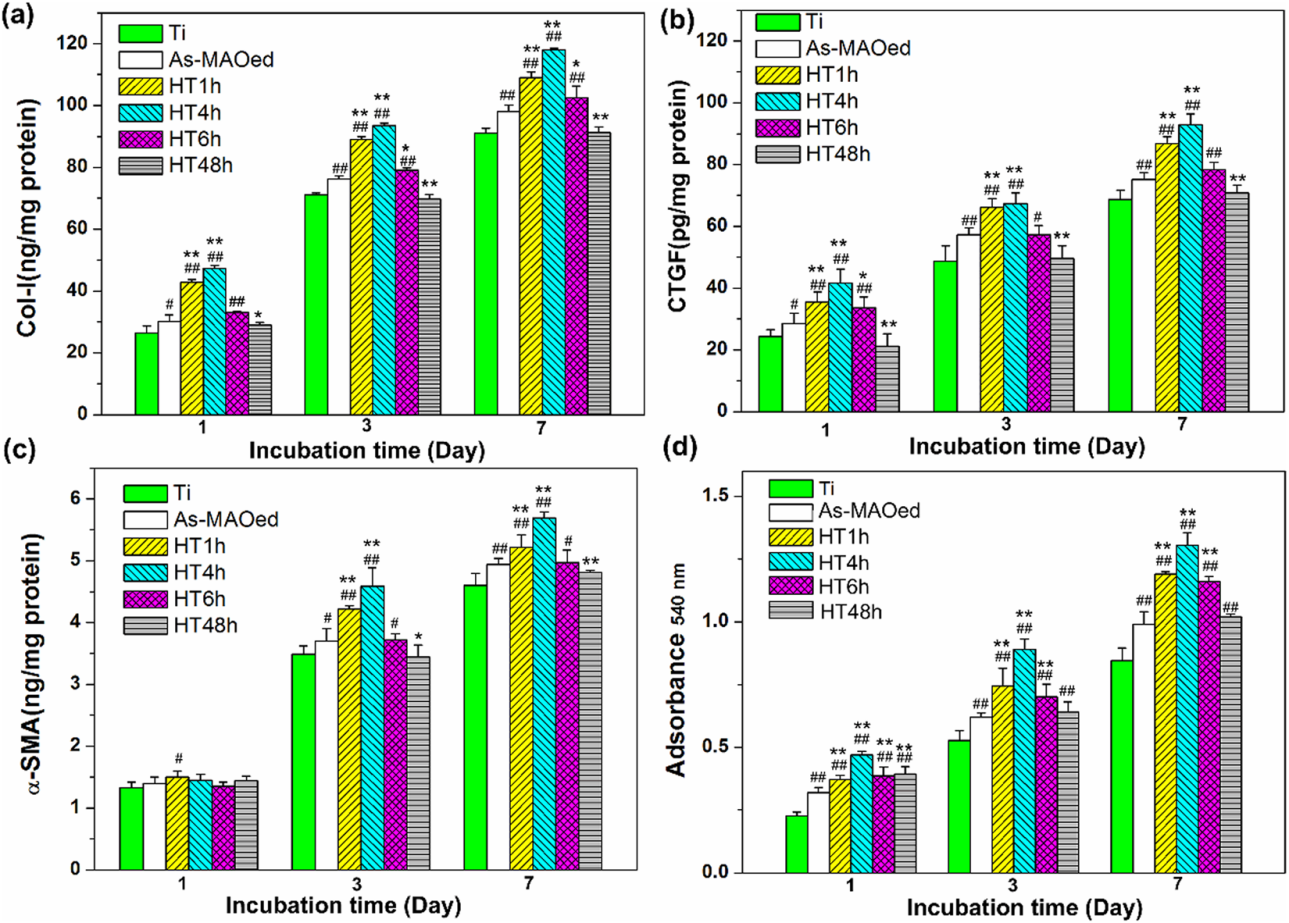
Intracellular protein contents of (**a**) Col-I, (**b**) CTGF, (**c**) a-SMA and collagen secretion (**d**) by fibroblasts on different surfaces for 1, 3 and 7 days of incubation. ^#^p < 0.05 and ^##^p < 0.01 compared with Ti, ^*^p < 0.05 and ^**^p < 0.01 compared with as-MAOed TiO_2_.

Intracellular Col-I, CTGF and α-SMA synthesized in cells on different samples after 1, 3 and 7 days of culture are shown in Fig. 5(a,b and c), respectively. With the increased culture time from 1 to 7 d, the amounts of Col-I and CTGF for each sample increase, and at each time point, they have the similar trends: HT4h > HT1h > HT6h > As-MAOed TiO_2_ > HT48 ≈ Ti (Fig. 5(a) and (b)). For α-SMA, after incubation for 1 d, there are no highly significant differences in the content on different surfaces; with incubation time prolonged to 3 and 7 d, the amounts on all samples obviously increase and follow the order: HT4h > HT1h > HT6h ≈ As-MAOed TiO_2_ > HT48h ≈ Ti (Fig. 5(c)). The amounts of extracellular collagen secreted by fibroblasts on Ti, as-MAOed and HTed coatings after incubation for 1, 3 and 7 d were quantized in Fig. 5(d). After incubation for 1 d, compared to Ti, as-MAOed and HTed surfaces can promote the secretion of collagen, especially for HT4h. With incubation prolonged to 3 and 7 d, the secretion of collagen on each sample increases, following the order: HT4h > HT1h > HT6h > As-MAOed TiO_2_ ≈ HT48h > Ti.

### *In vitro* antibacterial activity evaluation

**Figure 6:**
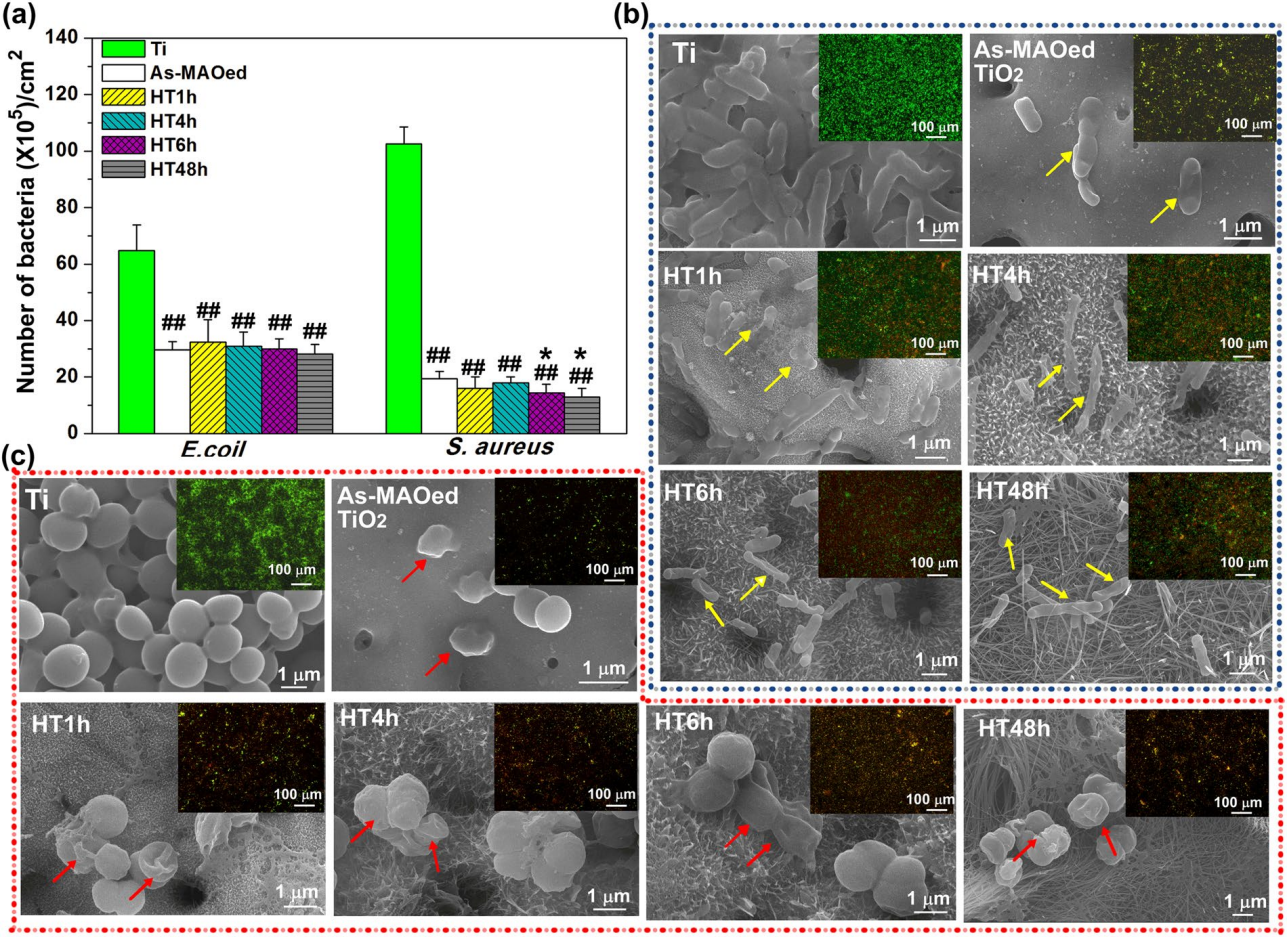
(**a**) Numbers of *E.coil* and *S. aureus* on different samples after 24 h incubation; SEM images and corresponding live-dead assay (insets) of *E.coil* (**b**) and *S. aureus* (**c**) cultured on different surfaces after 24 h incubation. ^##^p < 0.01 compared with Ti, ^*^p < 0.05 compared with as-MAOed TiO_2_.

The contact-killing activities of different surfaces were determined by evaluating the adhered bacteria after 24 h of incubation in Fig. 6(a). The adhered numbers of bacteria are obviously decreased on coating surfaces compared with those on Ti, especially for *S. aureus*. The average numbers of *E. coli* for coating surfaces are almost the same, about 30 × 10^5^ cm^−2^. There are no highly statistic differences of *S. aureus* numbers on HT1h, HT4h compared with that on as-MAOed TiO_2_, whereas the number on HT48 is lower than on other coatings. The bacteria on different surfaces after incubation for 24 h are further observed by SEM and live/dead stain assay in Fig. 6(b) and (c). *E. coli* are rod-shaped and undamaged binary fission when cultured on Ti, while many of them look corrugated and merged into the as-MAOed or HTed coatings (marked with yellow arrows in Fig. 6(b)). Similarly, *S. aureus* display round, smooth and intact surfaces on Ti, however, many cell debris and lysed cells are noticed on as-MAOed and HTed surfaces (marked with red arrows in Fig. 6(c)). The distributions and viabilities of *E. coli* and *S. aureus* adhered on different surfaces by live/dead strain are inserted in the corresponding SEM images. Most of *E. coli* and *S. aureus* on Ti are live and stained in green, while the green fluorescence intensities decrease obviously on as-MAOed and HTed surfaces compared with those on Ti. On the same coating, the green fluorescence intensity of *E. coli* is stronger than those of *S. aureus*, which is congruent with the counting results in Fig. 6(a), indicating that *S. aureus* is more sensitive to the coatings.

## Discussion

Micro-arc oxidation could form nanograined, microporous, adhered and ion-doped oxide films on Ti surfaces^15,16,23^. MAO followed by HT has been widely applied to fabricate nanostructured hydroxyapatite layer to improve osteointegration of titanium alloys, and its mechanism has been well explored^17,18^. For HTO, Wei *et al*. recently fabricated its nanorod arrays on amorphous TiO_2_, and the incorporated elements of Ca, P, Si and Na, formation of anatase layer on amorphous TiO_2_ as well as corrosive attack of OH groups are reported the key factors for the formation of HTO^15^. As-MAOed TiO_2_ doped with Zn and P herein is consisted of nanograined anatase and rutile, so the formation process of HTO should be not completely consistent with that by Wei *et al*.^15^. Combined previous reports^27–30^ with the results in Figs 1 and 2, formation process of HTO can be analyzed as follows. During HT process, Ti-O-Ti bonds initially dissolve due to the attack of OH^−^^27,29^, following the reaction: TiO_2_ + OH^−^ → HTiO_3_^−^. Then, HTiO_3_^−^ reacts with TiO_2_ and H_2_O to form HTO nuclei: 3TiO_2_ + 2HTiO_3_^−^ + 2H_2_O → H_2_Ti_5_O_11_·H_2_O + 2OH^−^. Afterwards, for anatase, exfoliations of single-layered TiO_6_ octahedra formed by the attack of OH^−^, sharing four edges with HTO, can assemble themselves on the template of HTO^28–30^. However, for rutile which is composed of TiO_6_ octahedrons sharing two edges with HTO, the formation of HTO should be ascribed to a total structural destruction and recrystallization^29^. With the prolonged HT, attack and dissolution of TiO_2_ layer continue, thinning the TiO_2_ layer (Fig. 2), and the HTO nuclei eventually grow along c-axis into nanorod and nanofibre structure (Figs 1 and 2), owing to the epitaxial crystal growth^15^. Moreover, during this process, Na^+^ ions from the HT solution should join in the interlayer space to neutralize the negative charge of TiO_6_, resulting in the doping of Na in HTO (Fig. 1).

It is known that surface properties including ion releasing, phase/chemical composition, roughness, nano-topography and hydrophilicity *et al*. affect the response of cells. As-MAOed and HTed coatings have the similar phase composition, roughness and wettability (Figs 1–3 and Supplementary Figure S1), except the nano-topography and amount of Zn. Zinc plays an important role in the complex regulation of signal molecules and mediators, for example the cytokines and growth factors, which then enable tissue regeneration in injured tissue^19–22^. Zn should contribute to the improved responses of L-929 on as-MAOed TiO_2_ herein. For HTed coatings (e.g.HT1h, HT4h and HT6h), compared with as-MAOed TiO_2_, the amounts of Zn are much lower, whereas, the adhesion and proliferation of fibroblasts are further enhanced (Fig. 4). It is known that protein adsorbed on surfaces determines cell adhesion^18,31^. Compared with other HTed surfaces, HT48h has more total protein, whereas its mitochondrial activity and viability of L-929 are obviously weaker (Fig. 4(a)). It indicates that nano-topology of HTO should be the key factor which leads to the different adhesion and proliferation of cells. The geometric parameters of nano-topology include diameter, orientation and inter-space, etc. It is widely reported that inter-space is efficient in affecting the formation of focal adhesions, which mediate cell response to materials^18,31,32^. As explored in previous studies, the formation of focal adhesion gave rise to be enhanced on the <96 nm spaced nanorods, and more significant with those of mean 67 nm, while inhibited on the >137 nm patterned nanorods, resulting in different response of cells^18,26^. The average inter-spaces of nanoplates, nanorods and nanofibres are 45, 63 and 97 nm for HT1h, HT4h and HT6h, respectively, which should be benefit for the formation of focal adhesion. Subsequently, the adhesion, proliferation and intracellular proteins synthesis (Col-I, CTGF and a-SMA) on the nanoplates or nanorods are enhanced, especially on HT4h which has a 63 nm inter-rod spacing (Figs 1 and 5). HT48h has a slightly larger inter-space (105 nm) of nanofibres compared with other HTed surfaces, resulting in a relatively weaker response of L-929 (Figs 1, 4 and 5). However, compared with Ti, the adhesion and proliferation of L-929 on HT48h are still enhanced, and it should be due to a comprehesive effect of surface properties (e.g. phase/chemical composition, super hydrophility, nano-topography) as discussed above. Moreover, extracellular collagen secretion are accelerated on HTed surfaces, indicating the remolding of collage matrix can be speeded up, especially on HT4h.

The antibacterial activity of coatings was evaluated using both *E. coli* (Gram-negative bacteria) and *S. aureus* (Gram-positive bacteria) colonies. It is known that infections associated with implants are mainly ascribed to adherent bacteria, and if bacteria become firmly attached and form a biofilm, they can withstand host immune responses. Compared with Ti, as-MAOed and HTed surfaces obviously reduce bacteria adhesion, especially for *S. aureus* (Fig. 6). It is known that Zn^2+^ shows antibacterial property through a mechanism of damaging bacterial cell membranes by the generation of reactive oxygen species^23^. As-MAOed TiO_2_ shows well antimicrobial activity against *E. coli* and *S. aureus*, and it should be ascribed to the high content of doped Zn^2+^. However, during the HT process, a large amount of Zn migrated out from TiO_2_ layer, resulting in low-dose of Zn contained in HTed coatings. Although the reported studies about effect of nano-topography on the bacteria adhesion are in controversial^24–26^, compared with as-MAOed TiO_2_, the nanostructured HTO herein (e.g. nanoplates, nanorods and nanofibres) can inhibit the adhesion and proliferation of *E. coli* and *S. aureus* in the similar degree. Furthermore, nanorods and nanofibres with relatively larger interspaces (e.g.HT6h and HT48h) herein are more efficient in inhibiting adhesion and reproduction of *S. aureus* (Fig. 6) compared with other HTed surfaces. It indicates that nano-topography of HTed surface should play a key role in inhibiting the bacterial adhesion. The exact mechanism of bacteria responding to nano-topography needs to be further explored.

In conclusion, nanoplate/nanorod/nanofibre H_2_Ti_5_O_11_·H_2_O outlayered Zn-doped TiO_2_ composite coatings were fabricated by a hybrid process of micro arc oxidation and hydrothermal treatment. During HT, TiO_2_ reacted with OH^−^ and H_2_O, resulting in the nucleation of HTO. With HT prolonged, HTO nuclei grew to nanorods and nanofibres, and the orientation of nano-rods/fibers also changed be quasi-vertical and parallel to Ti substrate, respectively. Compared to Ti, the adhesion and proliferation of fibroblasts were enhanced on as-MAOed TiO_2_ and HTed coatings; the phenotype, differentiation and extracellular collagen secretion were accelerated on vertical nanorods, especially on HT4h surface; HTed coatings all showed well antibacterial properties. Compared with Zn ions, nano-topography plays a key role in enhancing the responses of fibroblasts and antibacterial activity. Our study provides potential coatings (e.g. HT1h, HT4h) applied for percutaneous implant surface, and their fibroblast functions and anti-bacterial ability can be simultaneously enhanced.

## Methods

### Materials preparation and characterization

Ti plates (ϕ 14 mm) were MAOed at a voltage of 480 V and pulse frequency of 500 Hz for 2 min in an aqueous electrolyte containing 0.2 M zinc acetate, 0.02 M β-glycerophosphate disodium. Then, as-MAOed samples were HTed in 1 M NaOH at 200 °C for 1, 4, 6 and 48 h, and the obtained were referred to as HT1h, HT4h, HT6h and HT48h, respectively.

The phase compositions and morphologies of the coatings were examined by X-ray diffractometer (X’Pert PRO, Netherland), field emission scanning electron microscope (FESEM; SU6600, Hitachi, Japan), transmission electron microscopy (TEM; JEOL JEM-2000FX, Japan), and laser scanning confocal microscope (VK-9710, KEYENCE, Japan), respectively.

The adhesion strengths and wettability of coatings were measured by an auto scratch coating tester and surface contact-angle measurement machine (DSA30, Kruss, Germany), as detailed in previous work^16^.

*In vitro*, Zn and Ti ions released from coatings were measured by immersing the samples in 10 ml 0.9 wt% NaCl at 36.5 °C for 1, 7 and 14 days, and evaluating the accumulated concentrations by inductively coupled plasma emission spectroscopy (ICP-AES; Perkin Elmer, Optima 3000 DV, USA).

### Cytocompatibility and *in vitro* fibroblast response evaluation

#### Protein adsorption assay

For protein adsorption assay, each sample was cultured in 500 μl culture medium containing 90% α-MEM (Thermo Scientific, USA) and 10% fetal bovine serum (FBS; Thermo Scientific, USA) at 37 °C for 1, 4 and 24 h, respectively. Then, the absorbed proteins were detached by 250 µL of 1% sodium dodecyl sulfate (SDS) solution and determined by a MicroBCA protein assay kit (Pierce). Four samples of each group were tested (n = 4).

#### Cell culture

Mouse fibroblasts (L-929) from Institute of Biochemistry and Cell Biology of Chinese Academy of Sciences (Shanghai, China), were cultivated in a humidified atmosphere incubator with 5% CO_2_ at 37 °C. The culture medium contains 90% α-MEM, 10% fetal bovine serum, 1 mM sodium pyruvate (Sigma, USA) and 15 mM NaHCO_3_, respectively. It was refreshed every two days.

#### Cell adhesion and proliferation assessment

L-929 cells with a density of 2 × 10^4^ cells per cm^2^ were seeded on each sample and incubated for 1 h, and 1, 3, and 7 days. After each incubation period, the adhesion and proliferation of L-929 were evaluated by MTT assay, and absorbance was measured by a Multiscan Spectrum (Multiskan FC; Thermo, America). Four specimens for each group were tested.

After incubation for 1 and 3 days, cells were washed with phosphate buffered saline (PBS), fixed with 2.5% glutaraldehyde, dehydrated in graded ethanol, dried in vacuum overnight, and finally coated with gold to be observed by FESEM. The viability of cells were also evaluated by a live/dead viability/cytotoxicity kit (Invitrogen, Eugene, OR) and finally observed by an Olympus BX52 microscope.

#### Synthesis of Col-I and intracellular specific proteins

L-929 cells with a density of 4 × 10^4^ cells per cm^2^ were seeded on each sample and incubated for 1, 3, and 7 days. After washed by PBS, cells were lysed in 0.1 vol% Triton X-100 through five standard freeze-thaw cycles. The suspensions were centrifuged and supernatants were collected to evaluate intracellular contents of Col-I, CTGF and a-SMA by using ELISA kits (R&D,USA)^7^. Finally, the results were normalized to the intracellular total protein content. Four samples for each group were tested, and each test was repeated four times (n = 4).

#### Collagen secretion of fibroblasts

After 1, 3 and 7 days of culture, the cell-seeded samples were fixed in 4% paraformaldehyde for 0.5 h, stained in saturated picric acid solution containing 0.1% Sirius Red (Sigma, USA) for 18 h, washed with 0.1 M acetic acid, eluted by a destaining solution (0.2 M 1:1 NaOH/methanol), and finally measured by a spectrophotometer at 540 nm. Three samples for each group were tested.

### *In vitro* antibacterial test

Gram-positive *S. aureus* (ATCC 25923) and gram-negative *E. coli* (ATCC 25922) were employed and inoculated twice in nutrient agar to obtain bacteria in midlogarithmic phase of growth. Then they were adjusted to a concentration of 10^5^ CFU mL^−1^ in nutrient broth. Samples were placed in the centre of 24-well plates, and then added 1 mL of the above bacterial suspension. After incubated at 37 °C for 24 h, samples were washed by PBS for three times, and ultrasonically treated in 1 mL PBS at 40 W for 5 min. The obtained bacteria in suspensions were resampled to count the viable bacteria according to the National Standard of China GB/T 4789.2 protocol. Four samples from each group were tested and each test was repeated four times (n = 4). For the Live/Dead staining, the bacteria on each substrate after 24 h of culture were washed by PBS and stained by a Live/Dead® Baclight™ Bacterial Viability Kits (L13152) according to the operation instruction, and examined by an Olympus BX52 microscope. Furthermore, the morphologies of bacteria on different samples were observed by FESEM, and the specimens were prepared using the same procedures as described in Section 4.2.3.

### Statistical Analysis

SPSS 14.0 software (SPSS, USA) was used to analyze the data. A one-way ANOVA followed by a Student-Newman-Keuls post hoc test was used to determine the level of significance. p < 0.05 was considered to be significant, and p < 0.01 was considered to be highly significant. All the data are expressed as mean ± standard deviation.

## Electronic supplementary material

supplementary information
