# Supplementary material for: Cytocompatibility and antibacterial activity of nanostructured H2Ti5O11·H2O outlayered Zn-doped TiO2 coatings on Ti for percutaneous implants
Source: Sci Rep. 2017 Oct 24;7:13951. doi: 10.1038/s41598-017-13954-4 (PMC5654996; doi:10.1038/s41598-017-13954-4)
Supplement: Supplementary file 1 — supplementary information [file 41598_2017_13954_MOESM1_ESM.doc]

[**Cytocompatibility**](javascript:void(0);) **and** [**antibacterial**](javascript:void(0);) **activity of**

**nanostructured H_2_Ti_5_O_11_·H_2_O outlayered Zn-doped TiO_2_ coatings on** **Ti for percutaneous implants**

Lan Zhang, Juan Zhang, Fang Dai, Yong Han[[1]](#footnote-2)*


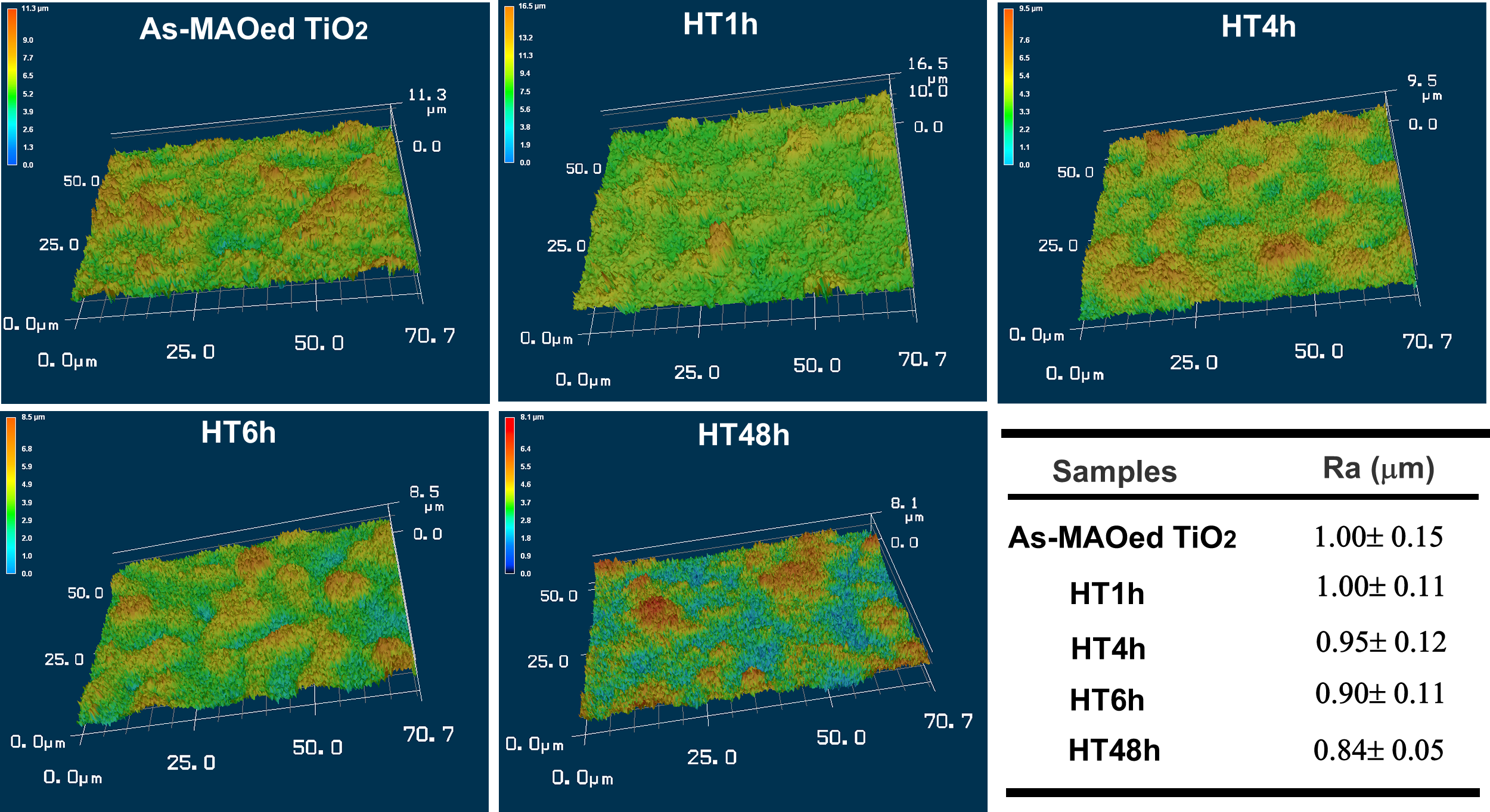


**Supplementary Figure S1** Laser confocal images and Ra values of different surfaces.


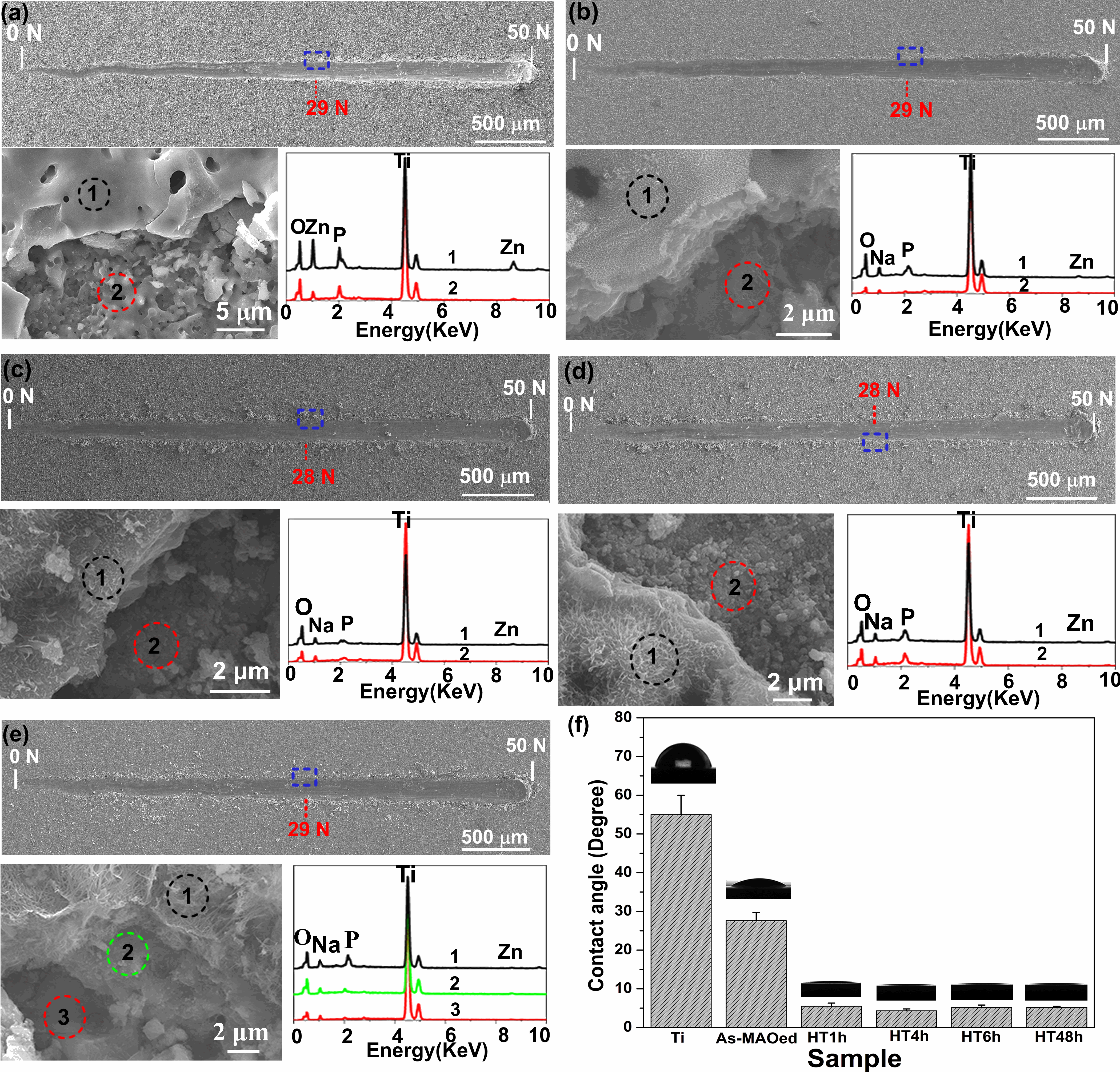


**Supplementary Figure S2** (a-e) Scratch morphologies of different coatings, together with amplified views of the initial failures: (a) As-MAOed TiO_2_, (b) HT1h, (c) HT4h, (d) HT6h and (e) HT48h; the corresponding EDX spectras were detected on surface (marked with 1) and delaminating regions (marked with 2 and/or 3); (f) contact angles of different samples.

1. ^^ Corresponding author, e-mail: yonghan@mail.xjtu.edu.cn, Tel.:+86 02982665580; fax:,+86 02982663453 [↑](#footnote-ref-2)
